# Application of the ASVCP guidelines for the establishment of haematologic and biochemical reference intervals in Icelandic horses in Austria

**DOI:** 10.1186/s13028-015-0120-4

**Published:** 2015-06-14

**Authors:** Ernst F. Leidinger, Judith Leidinger, Julia Figl, Bettina Rumpler, Ilse Schwendenwein

**Affiliations:** In Vitro Veterinary Laboratories, Rennweg 95, 1030 Vienna, Austria; Tierärztliche Praxis, Heiligenbrunnerg. 19, 7221 Marz, Austria; Clinical Pathology Platform, University of Veterinary Medicine, Veterinärplatz 1, 1210 Vienna, Austria

**Keywords:** Breed, Advia 2120i™, Dimension ExL™, Reference population, Preanalytics, Comparison

## Abstract

**Background:**

Despite the increasing popularity of Icelandic horses, published reference intervals (RIs) in this breed are rare. Due to their isolation and their small gene pool, alterations in some variables are likely and some possible breed-specific peculiarities have been described. The purpose of the present study was the establishment of comprehensive RIs in Icelandic horses according to recently published guidelines.

In a prospective observational study, blood samples were collected from the jugular vein of 142 Icelandic horses into EDTA and serum tubes. Reference intervals were established for haematologic and biochemical analytes on the Advia 2120i™ and the Dimension ExL™ by established methods. RIs were defined as central 95 % intervals bounded by the 2.5th and 97.5th percentiles with their 90 % confidence intervals, calculated according to recently published ASVCP guidelines. An inhouse-developed quality control system using observed total allowable error was used for the surveillance of the internal quality control preceding the measurements.

**Results:**

The RIs were as follows: haematocrit: 0.29–0.39, RBC: 5.79–8.63 T/l, haemoglobin: 102.0–142.3 g/l, MCV: 42–51 fl, platelets: 146–263 G/l, WBC: 4.13–8.57 G/l, segs: 1.98–4.73 G/l, lymphocytes: 1.25–3.49 G/l, monocytes: 0.06–0.31 G/l, eosinophils: 0.04–0.50 G/l, glucose: 4.0–5.7 mmol/l, urea: 3.2–6.4 mmol/l, creatinine: 79.6–141.4 μmol/l, total protein: 54.4–72.9 g/l, albumin: 27.7–36.8 g/l, total bilirubin: 8.1–21.1 μmol/l, triglycerides: 0.03–0.44 mmol/l, cholesterol: 1.75–2.90 mmol/l, ALP: 1.35–3.55 μkat/l, AST: 4.52–8.80 μkat/l, GLDH: 0.0–0.18 μkat/l, GGT: 0.11–0.39 μkat/l, CK: 2.53–6.52 μkat/l, LDH: 3.32–7.95 μkat/l, iron: 16.4–39.9 μmol/l, calcium: 2.69–3.19 mmol/l, phosphate: 0.5–1.3 mmol/l, magnesium: 0.6–0.9 mmol/l, sodium: 134–141 mmol/l, potassium: 3.6–4.7 mmol/l, chloride: 100–105 mmol/l.

**Conclusions:**

Reference intervals of several haematologic and biochemical analytes differed from the transferred historical reference intervals applied to equine samples in the authors’ laboratory. These might be of clinical importance in some analytes such as creatine kinase.

**Electronic supplementary material:**

The online version of this article (doi:10.1186/s13028-015-0120-4) contains supplementary material, which is available to authorized users.

## Background

Icelandic horses are small, long-living and robust. The average height at withers of the modern type is 136.4 cm (13.4 hands), established by video-morphometry [1]. They were bred completely isolated from other horse breeds for about a millennium and even today horses that have left Iceland are legally prevented from returning [2]. During the last four decades Icelandic horses have become increasingly popular as leisure and competition horses in many countries. The Icelandic horse studbook, ‘WorldFengur’, recorded more than 240,000 living Icelandic horses by October 2013 [3]. Intense selective pressures applied over time have resulted in substantial variation among horse breeds [4]. Statistically significant alterations of several haematologic and biochemical analytes have been described [5–7] and, therefore, divergent breed-related reference intervals (RIs) are to be expected [8, 9]. For example, Icelandic horses reportedly have the lowest number of monocytes of all horse breeds (1.7–5.4 % or 0.10–0.45 G/l) according to a recent study performed on the ADVIA 2120i haematology analyser [5]. Some studies found muscle enzyme activities that were significantly higher than in the general horse population [5, 10].

Unkel calculated RIs for a number of biochemical analytes for Icelandic horses. The RIs, however, were based upon a 16–84 % interval which makes a comparison with more recently generated 95 % population-based RIs difficult [11–13].

Recently breed-specific RIs have been established for three different horse breeds using the same haematology analyser (ADVIA) and a different chemistry analyser [5].

The purpose of the present study was to establish RIs specific for Icelandic horses that might be useful in improving the interpretation of laboratory test results. Comprehensive RIs for horses, regardless of breed, are rarely found in the peer-reviewed literature. The RIs were established by following the recently published guidelines of the American Society for Veterinary Clinical Pathology (ASVCP) [14, 15] with regards to the selection of the reference population, pre-analytical conditions, quality control, analytical conditions and statistical methods.

## Methods

### Study population

The study population was recruited from 4 stables in eastern Austria. All Icelandic horses were registered in Worldfengur database (www.Worldfengur.com). They were kept in paddocks year around with free access to shelter in a covered area. Only horses of 3 years or more were selected as Islandic horses are typically introduced into riding stables at an age of 3 years. In addition the following *a priori* inclusion criteria were chosen: resident in Austria for more than 6 month, no gestation or lactation, and the absence of excessive exercise the day before the blood sampling. Clinical examination was based on visual examination, auscultation of the chest and heart and the body temperature. Blood was collected from 141 horses.

The study was approved by the Ethics Commission of the University of Veterinary Medicine, Vienna, Austria (license number 19/04/97/2013). The blood samples were taken with the owners’ informed consent.

### Specimen collection

Blood was collected for 2 days in February 2013 between 9:00 and 12:00 a.m., within 3 to 6 h after the morning feed. The left jugular vein was punctured by a 20 G hypodermic needle and blood was allowed to flow freely along the vessel wall into the vial up to the filling mark.

Blood for the complete blood counts was collected into 4 ml EDTA-K3 tubes (Vacuette, Greiner bio-one, Kremsmünster, Austria). Blood for biochemical analysis was collected into 10 ml serum tubes with a clot activator and separator gel (Vacuette, Greiner bio-one, Kremsmünster, Austria). Blood was allowed to clot for 30 min at room temperature and then spun down for 10 min at 4200 RPM (3800 × *g*, Universal 16, Hettich, Tuttlingen, Germany).

### Haematological quality control (QC) and analysis

All samples were analysed within 6 h of sampling.

The EDTA-samples were analysed on an ADVIA 2120i™ (Siemens Healthcare Diagnostics, Vienna, Austria) with the multi-species software (version 5.3.1.-MS). The ADVIA™ system has recently been evaluated for the use in horses [16].

Three levels of quality control materials provided by the manufacturer (ADVIA® 3 in 1 TESTpoint^TM^ Hematology-Controls, Siemens Healthcare Diagnostics Inc., Tarrytown, NY, USA) were analysed daily according to the lab’s working instructions. The units, methods used and the total observed error (TEo) are shown in Table 1. The TEo was compared to the total allowable error (TEa) [17] to determine whether the assay performance was satisfactory [18].

**Table 1:** Analytical methods used on the ADVIA 2120i

Analyte	Unit	Method	TEo (TEa) (%)
Haematocrit (Hct)	l/l	Calculated from Hct, MCV	3.7 (10)
Erythrocytes (RBC)	T/l	Light scatter	2.1 (10)
Haemoglobin (Hb)	g/l	Cyanide-free colorimetric method	1.9 (10)
MCV	fl	Light scatter/calculated	1.9 (7)
MCH	pg	Calculated	NA
MCHC	g/l	Light scatter/calculated	5.0 (7)
CH	g/l	Light scatter/calculated	NA
CHCM	g/l	Light scatter/calculated	NA
RDW	%	Light scatter/calculated	NA
Platelets (PLT)	G/l	Light scatter	11.1 (25)
MPV	fl	Light scatter	NA
WBC	G/l	MP stain, light scatter	10.2 (15)
MPXI	NU	MP stain, light scatter, calculated	NA
Automated differential	G/l	Light scatter	All within limits

*CH* cellular haemoglobin, *CHCM* corpuscular haemoglobin concentration, *Hct* haematocrit, *MCH* mean cellular haemoglobin, *MCHC* mean cellular haemoglobin concentration, *MCV*: mean cellular volume, *MP* myeloperoxidase, *MPXI* myeloperoxidase index, *NU* no unit, *NA* not applicable, *TEa* total allowable error, *TEo* total observed error, *RDW* red blood cell distribution width

The RIs and the confidence intervals (CIs) were also calculated for several ADVIA™-specific parameters: directly measured cellular haemoglobin (CH), directly measured mean corpuscular haemoglobin concentration (CHCM) and myeloperoxidase index (MPXI). In addition, RIs were also established for the Hct/Hb ratio.

### Biochemical QC and analysis

All samples were analysed within 6 h of sampling.

The serum samples were analysed on the fully selective wet chemistry analyser Dimension ExL™ (Siemens Healthcare Diagnostics, Vienna, Austria). Two levels of quality control material (Liquid Assayed Multiqual®, Bio-Rad Laboratories, Irvine, CA, USA, Cat. No. 694, 695) were analysed daily prior to running the samples. The units, methods and observed total error (TEo) results compared with the TEa are shown in Table 3 [19]. The reagents were ExL-specific (Siemens Healthcare Diagnostics, Vienna, Austria), with the exception of albumin and GLDH (Labor & Technik, Berlin, Germany). Enzymes were measured at 37 °C.

The units, methods used and the total observed error (TEo) are shown in Table 2.

**Table 2:** Analytical methods used on the Dimension ExL

Analyte	Unit	Method	TEo (TEa) (%)
Glucose	mmol/l	hexokinase-G6-PDG	5.0 (20)
Urea	mmol/l	enzymatic-kinetic UV-test with urease	13.1 (15)
Creatinine	μmol/l	picric acid	13.3 (20)
Total protein	g/l	biuret	9.9 (10)
Albumin	g/l	bromocresol green	10.8 (15)
Bilirubin, total	μmol/l	diazo-sulfaniline acid	11.1 (30)
Bilirubin, direct	μmol/l	diazo-sulfaniline acid/HCl	**37.3** (35)
Triglycerides	mmol/l	4-step, enzymatic	10.2 (25)
Cholesterol	mmol/l	cholesterol esterase/cholesterol oxidase	6.6 (20)
Alkaline phosphatase (ALP)	μkat/l (IU/l)	IFCC, kinetic	18.9 (25)
Aspartate aminotransferase (AST)	μkat/l (IU/l)	IFCC, with pyridoxale-phosphate activation	9.1 (30)
Alanine aminotransferase (ALT)	μkat/l (IU/l)	IFCC, with pyridoxale phosphate activation	23.0 (25)
Glutamate hydrogenase (GLDH)	μkat/l (IU/l)	DGKC, optimised	**45.1** (30)
γ-glutamyl transferase (GGT)	μkat/l (IU/l)	IFCC, kinetic	21.9 (20)
Lipase	μkat/l (IU/l)	DGGR	24.5 (25)
Creatine kinase (CK)	μkat/l (IU/l)	IFCC, kinetic	7.1 (30)
Lactate dehydrogenase (LDH)	μkat/l (IU/l)	IFCC, kinetic	8.7 (20)
Iron (Fe)	μmol/l	Ferene	6.3 (30)
Calcium (Ca)	mmol/l	α-cresolpthaleine	7.5 (10)
Phosphorus (P)	mmol/l	phosphate-molybtate, modified	10.2 (15)
Magnesium (Mg)	mmol/l	methylthymol blue	10.9 (20)
Sodium (Na)	mmol/l	ISE	3.3 (5)
Potassium (K)	mmol/l	ISE	**7.3** (5)
Chloride (Cl)	mmol/l	ISE	4.8 (5)

The TEo in bold indicates that the value exceeded the ASVCP-recommended TEa during the period of analysis

*DGKG* Deutsche Gesellschaft für klinische Chemie, *DGGR* 1,2-o-dilauryl-rac-glycero glutaric; acid-(6′-methylresorufin), *HCl* hydrochloric acid, *IFCC* international federation for clinical chemistry, *ISE* ion selective electrode, *PDG* pyruvate dehydrogenase, *TEa*: total allowable error, *TEo* total observed error

### Statistics

The reference intervals were calculated following the ASVCP guidelines [14]. Normality was assessed by the Anderson-Darling test. Outliers were identified by Tukey and Dixon-Reed methods and were eliminated after visual inspection. Data analysis was performed by MS Excel (Microsoft, Redmond, WA, USA) with the macro-instruction set Reference Value Adviser v1.3 (Ecole Nationale Vétérinaire, Toulouse, France) according to published guidelines [20]. Depending on the distribution, the parametric or robust method with or without Box-Cox transformation was applied to calculate the 95 % population-based RIs.

Descriptive statistics were calculated by Analyze-It for MS Excel (Analyze-It Software Ltd, Leeds, UK).

Following the recommendations by the Clinical Laboratory Standards Institute (CLSI) and the International Federation for Clinical Chemistry (IFCC) [21], CI should not exceed 0.2 times (or 1/5) the width of the RI (WCI/WRI <0.2, where WCI is the width of the CI and WRI is the width of the RI).

## Results

The median age of the reference animals was 13.7 years, the mean age 13.0 years and maximum age of 27 years. The population consisted of 88 geldings, 47 mares and 6 stallions. Two horses were excluded from the beginning due to elevated liver parameters.

### Haematology results

**Fig. 1:**
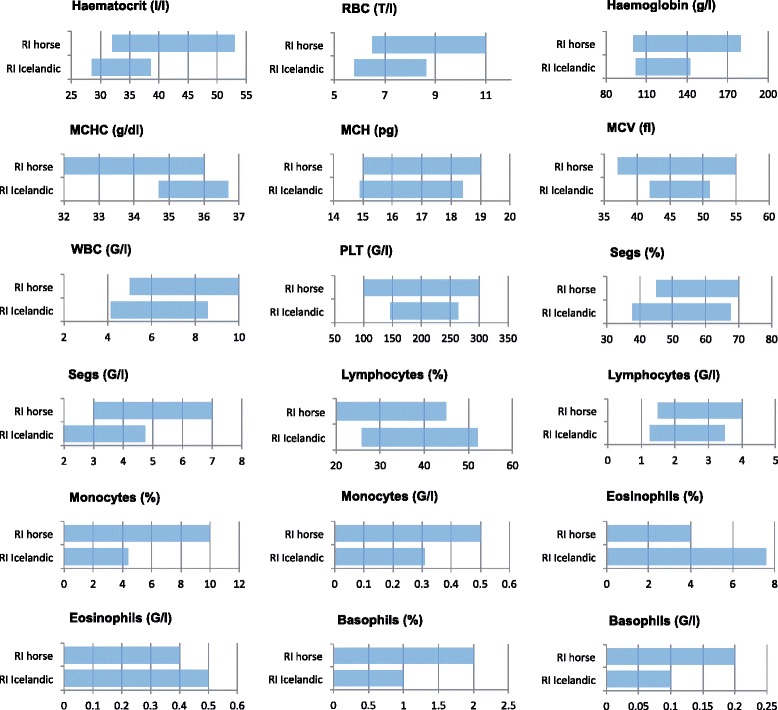
Comparison of the haematology RIs generally used for horses in the authors’ laboratory with the RIs established for Icelandic horses

All haematology analytes were calculated by the non-parametric method, because the required number of reference individuals (*n*≥120) was achieved. A normal distribution was observed with the following analytes: RBC, MCHC, MCH, RDW, MPV, Hct/Hb, and lymphocytes %. Results and their CIs are summarised in Table 3. In Fig. 1 the calculated RIs are compared to the haematology RIs used for horses in the author’s lab.

**Table 3:** Reference limits and their CIs for haematology analytes; n indicates the number of reference individuals after the removal of outliers, CI width describes the width of the lower and upper reference limit in comparison to the width of the RIs

Analyte	Number	Reference interval	CI lower reference limit	CI upper reference limit	CI width ratios
					LRL/URL
Haematocrit	132	0.29–0.39	0.29–0.29	0.38–0.40	0.0 / 0.2
RBC	136	5.79–8.63	5.48–5.98	8.46–8.68	0.2 / 0.1
Haemoglobin	134	102.0–142.3	100.0–103.0	137.0–145.0	0.1 / 0.2
Hct/Hb	131	2.72–2.88	2.69–2.74	2.87–2.89	**0.3** / 0.1
MCHC	137	347.0–366.6	346.0–349.0	364.0–369.0	0.2 / **0.3**
MCH	139	14.9–18.3	14.4–15.0	18.0–18.8	0.2 / 0.2
MCV	138	42–51	41–43	50–52	0.2 / 0.2
CH	137	151.9–184.1	148.0–155.0	181.0–187.0	0.2 / 0.2
CHCM	137	349.9–375.6	348.0–352.0	374.0–381.0	0.2 / 0.2
RDW	139	16.8–19.6	16.3–16.9	19.1–19.8	0.2 / **0.3**
WBC	138	4.13–8.57	3.63–4.23	8.20–8.83	0.1 / 0.1
Platelets	135	146–263	140–156	256–282	0.1 / 0.2
MPV	138	6.1–10.9	6.1–6.3	10.0–11.3	0.0 / **0.3**
Segs (%)	138	37.8–67.7	34.8–41.4	64.9–69.6	0.2 / 0.2
Segs (abs)	136	1.98–4.73	1.83–2.28	4.55–4.90	0.2 / 0.1
Lymphocytes (%)	136	25.7–52.1	21.4–27.4	50.9–54.2	0.2 / 0.1
Lymphocytes (abs)	132	1.25–3.49	1.01–1.40	3.19–3.70	0.2 / 0.2
Monocytes (%)	137	1.2–4.4	0.7–1.5	4.0–4.9	0.2 / 0.2
Monocytes (abs)	136	0.06–0.31	0.05–0.07	0.27–0.37	0.1 / **0.4**
Eosinophils (%)	124	0.7–7.6	0.5–1.1	7.4–8.1	0.1 / 0.2
Eosinophils (abs)	123	0.04–0.50	0.03–0.07	0.48–0.52	0.1 / 0.1
Basophils (%)	139	0.1–1.0	0.1–0.2	0.8–1.1	0.1 / **0.3**
Basophils (abs)	138	0.01–0.07	0.00–0.01	0.05–0.08	0.1 / **0.4**
Large unstained cells (%)	139	0.1–0.5	0.0–0.1	0.4–0.6	**0.3** / **0.5**
Large unstained cells (abs)	138	0.00–0.03	0.00–0.01	0.03–0.04	0.2 / **0.3**
MPXI	138	1.3–17.2	0.6–2.4	16.0–18.5	0.1 / 0.2

Bold: ratio exceeded the desired maximum ratio (<0.2)

*CH* cellular haemoglobin, *CHCM* corpuscular haemoglobin concentration, *CI(s)* confidence interval(s), *Hb* haemoglobin, *Hct* haematocrit, *LRL* lower reverence limit, *MCH* mean cellular haemoglobin, *MCHC* mean cellular haemoglobin concentration, *MPV* mean platelet volume, *MP* myeloperoxidase, *MPXI* myeloperoxidase index, *PLT* platelets, *RBC* red blood cells, *RDW* red blood cell distribution width, *RI(s)* reference interval(s), *URL* upper reference limit, *WBC* white blood cells

### Biochemistry results

**Fig. 2:**
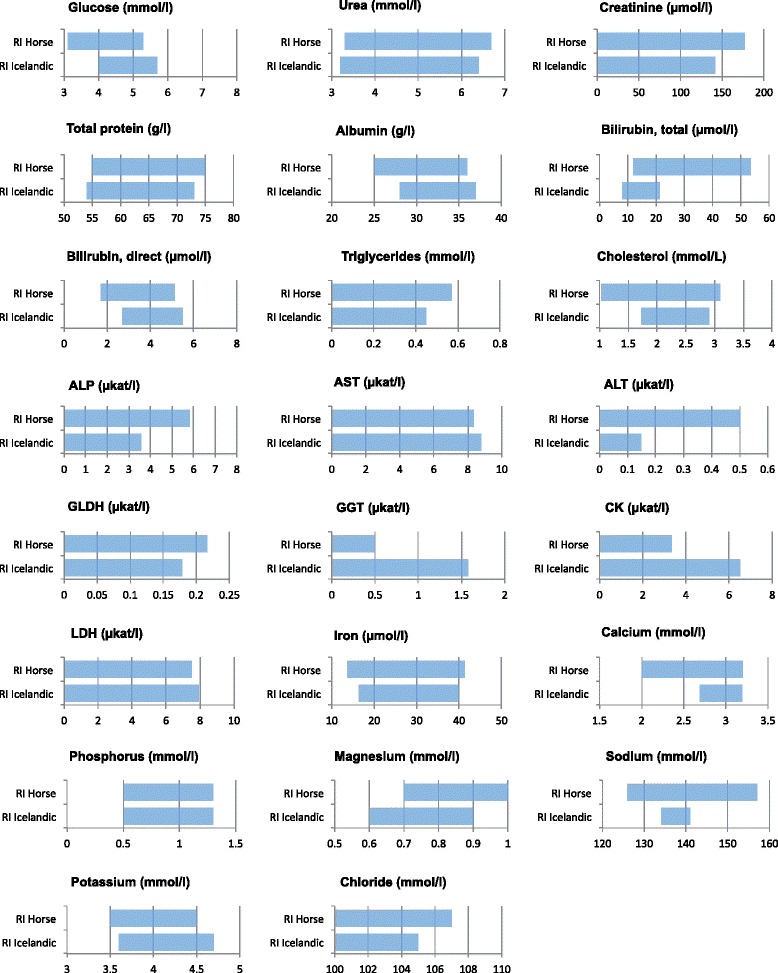
Comparison of the biochemistry RIs generally used for horses in the authors’ laboratory with the RIs established for Icelandic horses

The RIs and the 90 % CI of the upper and lower RL for all biochemical analytes except potassium were calculated by the non-parametric method, because the required number of reference individuals (*n* >120) was achieved. Potassium (*n* = 109) was calculated by the parametric method after Box-Cox transformation due to the large number of outliers which were excluded. The CIs were calculated by bootstrap. A normal distribution was observed with the following analytes: total protein, albumin, total bilirubin, triglycerides, cholesterol, AST, iron and calcium. The biochemistry RIs and their CIs are summarised in Table 4. In Fig. 2 the calculated RIs are compared to biochemistry RIs used for horses in the author’s lab.

**Table 4:** Reference limits and their confidence intervals for biochemical analytes; n indicates the number of reference individuals after the removal of outliers, CI width ratio describes the width of the lower and upper reference limit in comparison to the width of the RIs

Analyte	Number	Reference interval	CI lower reference limit	CI upper reference limit	CI width
					ratio
					LRL / URL
Glucose	135	4.0–5.7	3.9–4.1	5.5–5.8	0.1 / 0.2
Urea	139	3.2–6.4	3.0–3.5	6.0–6.7	0.2 / 0.2
Creatinine	137	79.6–141.4	79.6–88.4	132.6–141.4	0.1 / 0.1
Total protein	138	54.4–72.9	53.1–55.5	71.4–75.1	0.1 / 0.2
Albumin	132	27.7–36.8	27.3–28.5	35.4–37.2	0.1 / 0.2
Bilirubin, total	132	8.1–21.1	7.5–9.7	20.2–22.4	0.2 / 0.2
Bilirubin, direct	136	2.7–5.5	2.6–2.9	5.1–5.6	0.1 / 0.1
Triglycerides	133	0.03–0.44	0.01–0.06	0.38–0.47	0.1 / 0.2
Cholesterol	136	1.75–2.90	1.68–1.81	2.77–3.00	0.1 / 0.2
ALP	135	1.35–3.55	1.28–1.52	3.45–3.67	0.1 / 0.1
		(81–213)	(77–91)	(207–220)	
AST	133	4.52–8.80	3.85–4.82	8.15–8.87	0.2 / 0.2
		(271–528)	(231–289)	(489–532)	
ALT	138	0.01–0.15	0.0–0.02	0.15–0.17	0.1 / 0.1
		(0.5–9.0)	(0.0–1.0)	(9.0–10.0)	
GLDH	132	0.0–0.18	0.0–0.002	0.16–0.19	0.0 / 0.2
		(0.0–10.7)	(0.0–0.1)	(9.4–11.5)	
GGT	127	0.11–0.39	0.06–0.14	0.36–0.40	0.2 / 0.1
		(6.6–23.6)	(3.3–8.2)	(21.4–23,8)	
Lipase	135	0.43–1.12	0.38–0.47	1.07–1.15	0.1 / 0.1
		(26–67)	(23–28)	(64–69)	
CK	134	2.53–6.52	2.28–2.72	5.92–6.57	0.1 / 0.2
		(152–391)	(137–163)	(355–394)	
LDH	136	3.32–7.95	2.80–3.70	7.65–8.17	0.2 / 0.1
		(199–477)	(168–222)	(459–490)	
Iron (Fe)	137	16.4–39.9	14.7–21.7	38.0–41.4	**0.3** / 0.1
Calcium (Ca)	139	2.69–3.19	2.65–2.73	3.16–3.25	0.2 / 0.2
Phosphate (P)	139	0.5–1.3	0.4–0.6	1.3–1.5	**0.3** / **0.3**
Magnesium (Mg)	138	0.6–0.9	0.6–0.6	0.9–0.9	0.0 / 0.0
Sodium (Na)	138	134–141	134–135	140–141	0.2 / 0.2
Potassium (K)	109	3.6–4.7	3.5–3.6	4.6–4.8	0.1 / 0.2
Chloride (Cl)	133	100–105	100–100	105–105	0.0 / 0.0

Bold: ratio exceeded the desired maximum ratio (<0.2). Enzyme activities in brackets are in IU/l

*ALT* alanine aminotransferase, *AP* alkaline phosphatase, *ASVCP* american society for veterinary clinical pathology, *AST* aspartate aminotransferase, *CK* creatine kinase, *GGT* γ-glutamyl transferase, *GLDH* glutamate hydrogenase, *LDH* lactate dehydrogenase, *LRL* lower reverence limit, *RI(s)* reference interval(s), *URL* upper reference limit

## Discussion

The aim of this prospective study was to establish comprehensive RIs with well characterised analytical methods in a population of Icelandic horses with a well defined health state and in accordance with recently published guidelines [14, 15, 21]. Haematological and biochemical RIs may vary with age, sex, and season [5, 22]. However, data were not stratified in our study because under the conditions the horses were kept, a homogenous distribution for the variables mentioned above could not be achieved. The horses were at least 3 years of age and many more geldings were represented compared to mares. However this is representative of the overall Icelandic horse population in eastern Austria.

Furthermore, all horses in this study were registered in Worldfengur, where of approximately 35 % were imported from Iceland directly. It is also know from genetic studies that very little variation was found between Icelandic horses in different countries [23]. Therefore, the RI established in our study most likely are also representative for the Islandic horse population in general, despite the under-representation of mares.

In addition to a reliable reference population, the quality specifications for the methods are of paramount importance for the reliability of the RIs [24], although QC data are rarely included in reports of RI studies. In one recent study, mean and coefficient of variation of analytes were presented [25]. Friedrichs *et al.* recommended the recording of the TEo for all methods [15]. In our study, all haematology analytes achieved the quality goals (Table 1). The TEo of a given biochemistry analyte was compared to the TEa recommended by the ASVCP guidelines on total error and was for most methods below the recommended limit [14]. The biochemical analytes direct bilirubin, potassium, and GLDH did not meet the analytical goals. The deviations for direct bilirubin and potassium were small; in the case of potassium, the TEo deviation was caused by a single QC value exceeding the 2 standard deviation range. The TEo for GLDH requires a more detailed consideration: while a TEo of 30 % is typical for many enzymes, at a TEo of 45 %, a given value could be 45 % higher or lower than the true activity. However GLDH is a reliable indicator of hepatocyte damage, hence it was decided to include the RIs for GLDH, because there are few reference intervals for this analyte in the peer-reviewed literature. The RIs compared well to other published values [5], and the CIs were reasonably narrow. Another source for poor method performance of these few analytes might be the instability of the QC-material suspected in 1 level in this study due to trend for decreasing values in results over time.

The most noticeable finding in the haematologic raw data was the high number of outliers for the eosinophil percentage. The Dixon and Turkey tests identified 15/139 values as outliers. With two exceptions, all animals with increased eosinophil proportions were from the same riding stable. The reason for these outliers is not known. All horses were clinical healthy and regularly dewormed, but the increase could reflect a subclinical parasitism or allergy [26]. In one study, increased eosinophil numbers were found in horses kept outdoors 24 h a day [27].

To establish a diagnosis of eosinophilia as well as the increase of other leukocyte fractions, only the absolute numbers must be considered [28, Additional file 1]. The relative numbers, however, are still widely used among referral laboratories and clinicians [29]. They are applied for manual differentials and for the analysers’s internal quality control, and therefore reported in this study.

The RIs for Hct/Hb was included because it proved useful for the primary plausibility checks in our LIMS of haemograms measured on the ADVIA. According to our working instructions, a spun haematocrit is performed, if the ratio exceeds the lower or upper RL (unpublished data).

Haematologic RIs on the ADVIA for Icelandic horses were established in a previous study for 6 different horse breeds, including 84 Icelandic horses and used similar statistical methods [5]. While there was a good agreement in the overall data, differences were found for the following analytes: haematocrit, MCHC, MCV, WBC, platelets, lymphocytes (absolute) and eosinophils (relative and absolute). For MCHC, much higher ranges were found in that and another study [28] with reference intervals of 40.8 to 44.1 and 35 to 42 g/l, respectively. The reason for this is not clear. Our MCHC values compared well to the directly measured CHCM values.

The MPXI seemed to be useful as additional inflammatory parameter for the diagnosis of sepsis in horses [30], but this could not be confirmed in another study [31].

This low monocyte count found by Gieseler, was confirmed by our data from the ADVIA differentials [5]. It may, however, be caused by the way the ADVIA displays the monocytes of Icelandic horses in the Perox channel, rather than by a breed-associated peculiarity [32]. However, the routine gating is likely to be applied since the manual gating, while not difficult, is time consuming.

Analysis of potassium data revealed 30/139 (21.6 %) outliers, almost all of them from a single stable. The eliminated values exceeded the RIs given elsewhere [5]. Most likely this was caused by a pre-analytical problem, but a definitive reason for this finding was not identified. Potassium is contained in high concentrations in the equine RBCs, WBCs and PLT and can diffuse into serum [33]. The room temperature in the preparation room where the blood was left for clot formation could not be tightly controlled. No serum sample was visibly haemolysed, and no other analytes were affected.

In several publications, CK, AST and LDH activities were increased in Icelandic horses compared to standard and thoroughbred horses [10, 11, 34]. Other studies showed no increase in liver- and muscle-specific enzymes in horses in Iceland or at 24 weeks following their importation to Germany [35]. Our study provides support for elevated CK activity in Icelandic horses. The upper reference limit in Icelandic horses (6.52 μkat/l, CI: 5.92–6.15) was approximately two times higher than for the general horse populations and about three times higher than that found for horses in general in one study [6]. It compares well to several other studies [6, 10, 11]. It is, however, lower than the upper RL found by Gieseler with the same method (11.14 μkat/l) [4]. The reasons for these high CK activities are not clear, but an increased muscle mass in relation to the body mass [36], different serum isoenzyme CK patterns [37], or a tendency to form macroenzyme complexes that have a slower rate of elimination [38] could contribute to higher CK activities. Further studies are needed to clarify the cause(s).

The calculation of CI of the RLs should be part of the establishment of reference intervals [21]. CIs provide an estimate of the uncertainty of the limits and are generally narrower for large samples sizes [14]. For the non-parametric calculation of CI, at least 120 individuals are required, if less are available, other methods like bootstrap have to be used [14]. This was only necessary for potassium in our study.

The width of the 90 % CIs calculated in this study, is not described in detail in the ASVCP online guidelines, but is described in the CLSI guidelines [21] and by Friedrichs *et al.* [15]. The CI width exceeded 0.2 in 9/26 (34.6 %) of haematology analytes for at least one RL. This occurred most frequently with small fractions (differential cell count percentages), where effective outlier treatment was not possible. In other cases, the deviations are small and sometimes could be caused by the rounding of the figures (MCV).

In 2/24 (8.3 %) of biochemistry analytes the CI width exceeded the recommended value. In the case of phosphorus, both RLs, in the case of iron only the lower RL was affected. This could have been caused by the rounding of the figures within the LIMS. To improve the CI width and increase confidence in the validity of the reference interval, additional individuals could be added to the analysis.

The results highlight the difficulties of comparing the RIs generated in this study with other RIs published for Icelandic horses and for other breeds. These comparisons were complicated by the use of different analytical and statistical methods, as well as the different sizes and composition of the reference populations. The recently published guidelines for the establishment of RIs in veterinary medicine were comprehensive and could be easily applied. Following them as closely as possible, will facilitate the comparison of RIs in the future. Careful scrutiny of the sources of RIs and the reference population, collection conditions, analytical conditions and statistical methods is needed to determine if they may be suitable for transference validation and to determine if references intervals may need to be generated *de novo* for a particular breed and/or practice population.

Considering the tradition of veterinary laboratory testing it is amazing that somewhat arbitrarily established RI’s have been of clinical utility at all. Furthermore it is not clear if breed specific partitioning of RIs will improve clinical decisions and patient care as slight differences in RIs may not alter the clinical interpretation and decision-making process. Information on biologic variation that may help determine the significance of changes in patient data is scarce or absent for equine laboratory analytes. Only through continued experience with and analysis of data for breed-specific RIs will we be able to determine if breed-specific RIs may provide for improved clinical care for equine patients. The standardisation of the approach to reference interval generation, in accordance with the ASVCP guidelines, should help provide for a sound basis for comparisons of reference intervals. It will require time, however, for studies to be done using this standard approach.

## Additional file

Additional file 1: Figure S1. Additional material the example shows the differentials of 2 animals, both with 92 % neutrophils (segs) and 8 % lymphocytes. The first example with 4.0 G/l WBC results in an absolute number of neutrophils WRI and a lymphopenia. In the second example with 25.0 G/l WBC the result is a neutrophilia and an absolute number of lymphocytes WRI.

## Abbreviations

ALT: Alanine aminotransferase
AP: Alkaline phosphatase
ASVCP: American Society for Veterinary Clinical Pathology
AST: Aspartate aminotransferase
CH: Cellular haemoglobin
CHCM: Corpuscular haemoglobin concentration
CI(s): Confidence interval(s)
CK: Creatine kinase
CLSI: Clinical Laboratory Standards Institute
CV: Coefficient of variation
DGGR: 1,2-o-Dilauryl-rac-glycero glutaric acid-(6′-methylresorufin)
DGKC: Deutsche Gesellschaft für klinische Chemie
EDTA: Ethylenediaminetetraacetate
GGT: γ-glutamyl transferase
GLDH: Glutamate hydrogenase
Hb: Haemoglobin
Hct: Haematocrit
IFCC: International Federation for Clinical Chemistry
ISE: Ion selective electrode
LDH: Lactate dehydrogenase
LIMS: Laboratory Information and Management System
LRL: Lower reverence limit
MCH: Mean cellular haemoglobin
MCHC: Mean cellular haemoglobin concentration
MPV: Mean platelet volume
MP: Myeloperoxidase
MPXI: Myeloperoxidase index
PLT: Platelets
QC: Quality control
RBC: Red blood cells
RDW: Red blood cell distribution width
RI(s): Reference interval(s)
RL(s): Reference limit(s)
RPM: Rotations per minute
TEa: Total allowable error
TEo: Total observed error
URL: Upper reference limit
WBC: White blood cells
