# Supplementary figures and images for: Application of the ASVCP guidelines for the establishment of haematologic and biochemical reference intervals in Icelandic horses in Austria
Source: Acta Vet Scand. 2015 Jun 14;57(1):30. doi: 10.1186/s13028-015-0120-4 (PMC4466868; doi:10.1186/s13028-015-0120-4)

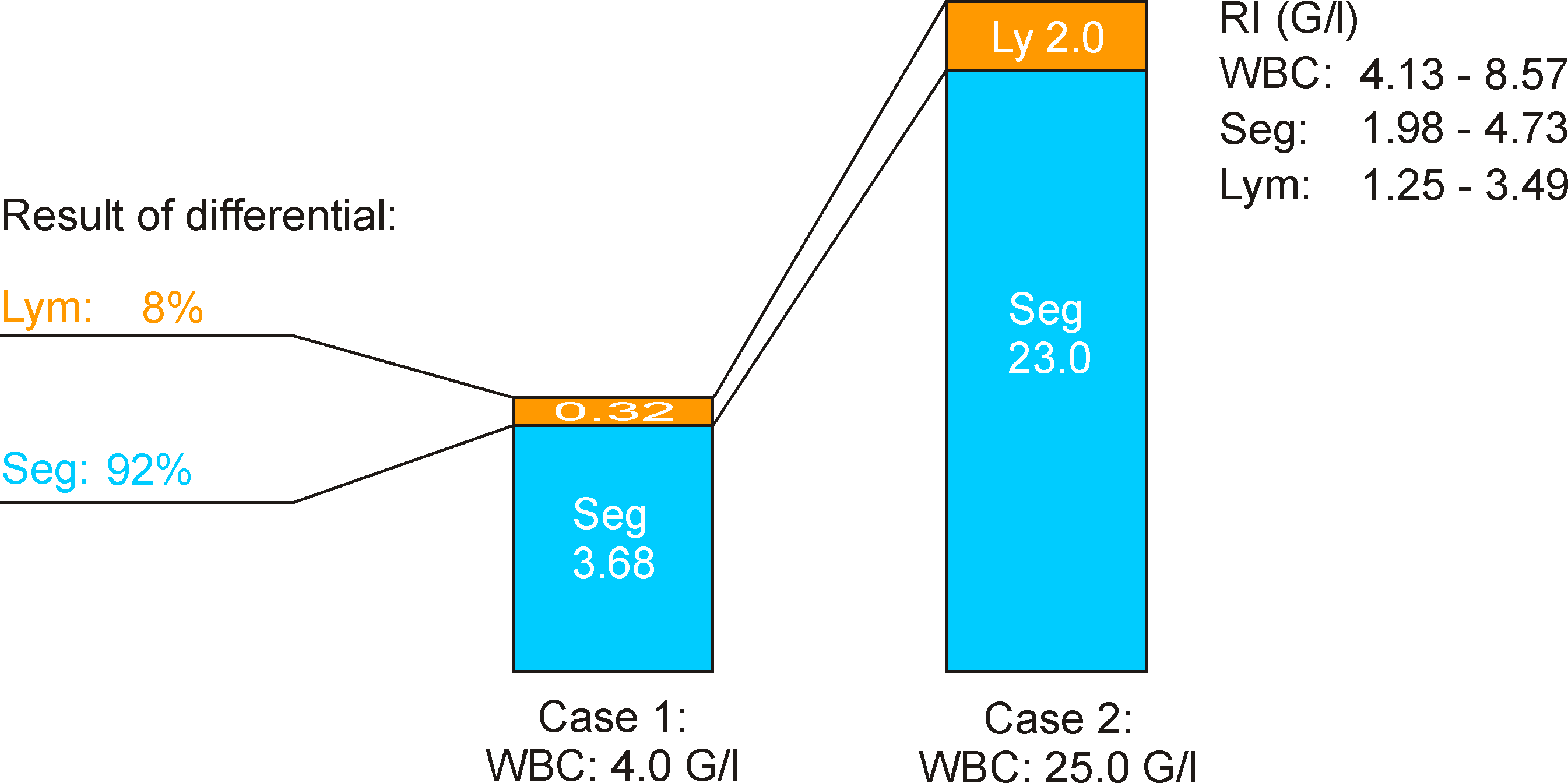

Supplement: Additional file 1: Figure S1. — Additional material the example shows the differentials of 2 animals, both with 92 % neutrophils (segs) and 8 % lymphocytes. The first example with 4.0 G/l WBC results in an absolute number of neutrophils WRI and a lymphopenia. In the second example with 25.0 G/l WBC the result is a neutrophilia and an absolute number of lymphocytes WRI. [file 13028_2015_120_MOESM1_ESM.png]
